# The insect repellent *N*,*N*-diethyl-*m*-toluamide (DEET) induces angiogenesis *via* allosteric modulation of the M3 muscarinic receptor in endothelial cells

**DOI:** 10.1038/srep28546

**Published:** 2016-06-27

**Authors:** Samuel Legeay, Nicolas Clere, Grégory Hilairet, Quoc-Tuan Do, Philippe Bernard, Jean-François Quignard, Véronique Apaire-Marchais, Bruno Lapied, Sébastien Faure

**Affiliations:** 1 University Bretagne Loire, Université d’Angers, UFR santé, département pharmacie, Angers, France; 2 GreenPharma S.A.S. 03 allée du titane, 45100 Orléans, France; 3 Inserm U1045-Centre de Recherche Cardio-Thoracique de Bordeaux, Université de Bordeaux, France; 4 Laboratoire des Récepteurs et Canaux Ioniques Membranaires (RCIM), UPRES-EA 2647, USC INRA 1330, Angers, France

## Abstract

The insect repellent *N,N*-diethyl-*m*-toluamide (DEET) has been reported to inhibit AChE (acetylcholinesterase) and to possess potential carcinogenic properties with excessive vascularization. In the present paper, we demonstrate that DEET specifically stimulates endothelial cells that promote angiogenesis which increases tumor growth. DEET activates cellular processes that lead to angiogenesis including proliferation, migration and adhesion. This is associated with an enhancement of NO production and VEGF expression in endothelial cells. M3 silencing or the use of a pharmacological M3 inhibitor abrogates all of these effects which reveals that DEET-induced angiogenesis is M3 sensitive. The experiments involving calcium signals in both endothelial and HEK cells overexpressing M3 receptors, as well as binding and docking studies demonstrate that DEET acts as an allosteric modulator of the M3 receptor. In addition, DEET inhibited AChE which increased acetylcholine bioavailability and binding to M3 receptors and also strengthened proangiogenic effects by an allosteric modulation.

The synthetic compound DEET (*N,N*-diethyl-*meta*-toluamide) is the most effective and widely used insect repellent^1^ in the world. The development and use of DEET to reduce disease transmission has undoubtedly increased patient survival since its introduction as an insect repellent. A small number of toxic side effects associated with the use of DEET in humans have been reported including seizures^2^ and Gulf War Syndrome^3^,^4^. Also, DEET may have carcinogenic properties, as have been found in human nasal mucosal cells^5^ or in Hodgkin lymphoma and soft tissue sarcomas^6^. Despite its discovery more than fifty years ago, the mode of action of DEET in insects has not been fully elucidated. In addition to the recent report showing that the neuronal insect ionotrope receptor Ir40a accounts for the widespread effect of DEET-induced repellency^7^, there is evidence that DEET inhibits acetylcholinesterase (AChE) activity in mosquitoes and humans^8^,^9^. AChE is not confined to the neuronal system and is expressed concurrent with acetylcholine receptors in a wide variety of cell types including epithelial, blood^10^, vascular endothelial (EC)^11^ and tumor cells^12^,^13^. The AChE inhibitor donepezil promotes angiogenesis^14^ both in EC and in a murine ischemic hind limb model. These findings have recently been confirmed by an *in vitro* study which shows that EC possess an autocrine non-neuronal cholinergic system that is able to regulate angiogenesis^15^.

Angiogenesis and vascular process are essential for tumor growth and metastasis^16^. Various cancers derived from epithelial cells^17^,^18^ express a cholinergic autocrine loop. Indeed, ACh secreted by tumor cells and neighboring cells interacts primarily with M3 muscarinic receptors expressed on tumor cells to stimulate tumor growth^19^. In non-small cell lung cancer, M3 receptor expression is associated with tumor progression and poor survival outcome^20^. Studies also show an increase of tumor angiogenesis through activation of M3 receptors in a mouse model of tumor-xenograft^21^,^22^. However, whether DEET interferes with angiogenesis is not known. The present study has been designed to: (i) establish the angiogenic properties of two relevant concentrations of DEET (10^−5 ^M, a plasma concentration common in exposed humans and 10^−8 ^M, a concentration found in surface water and wastewater)^23^,^24^ and (ii) determine the key targets involved in the control of this pathophysiological process with respect to the regulation of the M3 receptor.

## Material and Methods

### Ethical approval

All procedures involving animals, including the breeding protocols, were conducted in accordance with protocols approved by the ethical committee of the University of Angers and the regional ethics committee on animal testing. Furthermore, these experiments were approved by the ethical committee of the University of Angers and the regional ethics committee on animal testing (Authorization no. C49063, 11/22/2011). Furthermore, animal experiments were carried out in strict accordance with recommendations in the guidelines of the Code for Methods and Welfare Considerations in Behavioral Research with Animals (Directive 86/609EC).

### Cell culture

Human umbilical venous endothelial cells (HUVEC) obtained from male newborns were purchased from Lonza (CC2519) and grown in plastic flasks in EBM-2 medium (Lonza, CC3156) containing 1% L-glutamine (Lonza, BE17-605E), 1% streptomycin/penicillin (Lonza, DE17-602E) and 10% heat-inactivated fetal bovine serum (FBS, Gibco, 10270-106). HUVEC were used between the second and fourth passage. Cells were incubated for 24 h either in the absence or presence of DEET (Sigma–Aldrich, 1197007) used at the two concentrations 10^−8 ^M and 10^−5 ^M. In order to test the involvement of the M3 receptor, experiments were carried out either in the absence or presence of the selective M3 antagonist, *para*-fluorohexahydrosiladiphenidol (pFHHSiD, Sigma–Aldrich, H127) (pA2 = 7.9)^25^.

Primary EC were isolated from Swiss mice aortas. The extraction method was adapted from Kobayashi’s protocol^26^. Murine EC were cultured in growth medium EBM-2 supplemented with 5% heat-inactivated FBS and were used until their fourth passage.

Human embryonic kidney 293 (HEK-293) cells expressing recombinant G_αq/11_-coupled muscarinic M3 receptors (HEK-293/M3) were a generous gift from Dr Gary B. Willars^27^. These cells have been cultured in Dulbecco’s Modified Eagle Medium (DMEM, Lonza, BE12-604F), supplemented with 500 μg.mL^−1^ of geneticin (G-418) (Sigma–Aldrich, A1720), 1% of penicillin/streptomycin and 10% of heat-inactivated FBS.

U87MG glioblastoma-astrocytoma cells and B16F10 melanoma cells were a generous gift from Inserm 1066 MINT laboratory (Angers- France). These cells were cultured in DMEM, supplemented with 1% of L-glutamine, 1% of penicillin/streptomycin and 10% of heat-inactivated FBS.

### Proliferation assay of HUVEC and tumor cell lines

Effects of the two concentrations of DEET on HUVEC, U87MG or BF16F10 proliferation were analyzed by using CyQUANT Cell Proliferation Assay Kit (Molecular Probes, C7026). Briefly, 5.10^3^ cells per well were seeded into 96-well plates and allowed to attach overnight before being treated with DEET for 24 h. After growth medium removal, dye-binding solution was added into each microplate well and cells were incubated at 37 °C for 30 min. The fluorescence levels were detected on a Mithras LB940 (Berthold Technologies, Bad Wildbad, Germany) multimode microplate reader with filters for 485 nm excitation and 530 nm emission.

### Cell viability assay (MTT)

HUVEC were seeded at 10^4^ cells per well on 96-well plates. Cells were treated with DEET for 24 h. Viability was assessed by colorimetric analysis of MTT (Sigma-Aldrich, M5655). Absorbance values were obtained at a wavelength of 570 nm on a multimode microplate reader (Mithras LB940).

### Apoptosis measurement by flow cytometry

HUVEC were exposed either to DEET or actinomycin D, 1 μM as a positive control (Sigma–Aldrich, A9415) for 24 h and then fixed in 70% ethanol at 4 °C for at least 4 h. After a centrifugation at 1,500 × *g* for 5 min, cells were re-suspended in PBS containing 0.05 mg.mL^−1^ RNase (Sigma-Aldrich, R6513) and 10 μg.mL^−1^ propidium iodide (Sigma-Aldrich, P4170). Cellular DNA content was analyzed on a Cytomics FC500 MPL flow cytometer (Beckman Coulter, Villepinte, France). In all cases, 10,000 events were collected for analysis.

### RNA interference and transient transfection

To silence the M3 muscarinic acetylcholine receptor, siRNA duplexes specific for human M3 were obtained from Santa Cruz Biotechnology (SC35833). Transient transfection of HUVEC was performed according to the manufacturer’s protocol. Briefly, cells were seeded in six-well plates, grown for 24 h to 60% confluence and then transiently transfected with 10 nM of M3-specific or control siRNA using the transfection reagent provided, which also served as control without siRNA. Medium was replaced 24 h later by fresh medium and cells were grown for an additional 24 h.

### *In vitro* capillary network formation on ECM gel

HUVEC were treated with DEET for 24 h before being detached with trypsin EDTA acid. Cells were seeded at a density of 1.5 × 10^5^ cells per well. Each well was precoated with ECM gel (Sigma–Aldrich, E1270). Briefly, 150 μL of ECM gel was added into a 4-well plate and allowed to solidify for 1 h at 37 °C. Then, cells were incubated with medium containing 10% of FBS and allowed to adhere for 1 h after which the different stimuli were added. Tube formation was examined by phase-contrast microscopy (MOTIC AE21) after 24 h and the average length of capillaries was quantified using ImageJ software.

### *In vivo* ECMgel plug assay

All studies involving mice were in accordance with European guidelines and with the agreement of the regional veterinary services (Authorization no. C49063, 11/22/2011). Six week-old male Swiss mice were used.

Primary mouse EC were cultured in a 25 cm^2^ flask and were treated with DMSO or DEET for 24 h in the absence or presence of pFHHSiD (10^−7 ^M). After treatment, cells were detached and mixed with 500 μL of ECMgel with recombinant bFGF (300 ng.mL^−1^, Peprotech, 100-18B). This mixture was injected subcutaneously on the back of male Swiss mice. At day 14, ECMgel plugs were removed and homogenized in lysis buffer and incubated for 24 h at 4 °C and then disrupted with a Polytron (PRO250, Monroe, CT). Hemoglobin concentration was measured in the supernatants with Drabkin’s reagent (Sigma-Aldrich, D5941) according to the manufacturer’s instructions.

### Ectopic human glioma model in nude mice

Six week-old nude Swiss mice (Janvier SAS, Le Genest Saint Isle, France) were housed and maintained at the University animal facility. Tumor-bearing mice were prepared by injecting subcutaneously a suspension of 10^6^ U87MG glioblastoma-astrocytoma cells in 100 μL of DMEM solution into the right flank of athymic nude Swiss mice. From a volume of 100 mm^3^, mice were daily treated either by solvent, 10^−5 ^M DEET, 10^−7 ^M pFHHSiD or 10^−5 ^M DEET plus 10^−7 ^M pFHHSiD for 28 days. At the end of the protocol, mice were sacrificed and tumors were resected.

### Microvascular density (MVD) analysis

Tumors were included in paraffin blocks and sections of 5 μm were obtained with a microtome and deposed on polylysinated slides. Tumor sections were deparaffinized and endogenous peroxide production was inhibited with 1% hydrogen peroxide (Sigma Aldrich, 216763) for 15 min. Protein-protein links were lysed by 0.05% of pepsin in HCl 0.01 N for 30 min at 37 °C and nonspecific protein binding was blocked with 2% BSA for 30 min. 5 μm sections were incubated overnight at 4 °C with anti-mouse CD31 antibody (1:500 in BSA 2%, BD Bioscience, 557355). Sections were then incubated for 30 min with peroxidase-labeled biotinylated anti-rat antibody (1:100 in BSA 2%, Vector Laboratories, Vectastain) at room temperature for 30 min. Staining was amplified with avidin-biotin complex (Vector Laboratories, Vectastain) and signal was detected with diaminobenzidine (DAB) peroxidase substrate (Sigma-Aldrich, D12384) after an incubation of 10 min. Finally, sections were counterstained with hematoxylin for 20 s. Each tumor section was photographed 4 times by two independent manipulators with a white light microscope (objective X20).

### Biochemical assay of AChE activity

AChE activity was assayed using the Ellman’s method^28^. HUVEC (10^5^ cells per mL) were grown on 96-well plates for 24 h in presence of DEET at final dilution 10^−5 ^M or 10^−8 ^M. DEET-treated cells were then incubated for 60 min with 100 μL of 2 mM acetylthiocholine iodide (ATC, Sigma-Aldrich, A5751) and 100 μL of dithio-dinitrobenzoate (DTNB, 1 mM, Sigma-Aldrich, D8130) in 0.1 M phosphate buffer pH 8.1. AChE activity was measured at 37 °C by absorbance at 405 nm using a microplate reader (Mithras LB940). BW284c51 10^−5 ^M (Sigma-Aldrich) was used as control as acetylcholinesterase inhibitor. Results were expressed as a percentage of initial activity (without inhibitor).

### Adhesion assay on HUVEC

5.10^3^ cells per well were seeded into 96-well plates and were treated for 24 h with DEET. After incubation, the plate was shacked for 15 s. The supernatant with non-adherent cells was removed by three washes with washing buffer (0.1% BSA in medium without serum). Attached cells were fixed with 4% paraformaldehyde for 15 min at room temperature. Cells were rinsed two times with washing buffer, stained with crystal violet (1 mg.mL^−1^ in 2% of ethanol) for 10 min at room temperature and extensively washed with distilled water. Then, SDS 2% was added and incubated for 30 min at room temperature. Absorbance was then evaluated using a Mithras LB940 multimode microplate reader at 550 nm (Berthold Technologies).

### Migration assay

HUVEC were detached, washed twice in PBS and re-suspended in EBM-2 medium with 10% FBS. 5.10^4^ cells were seeded in the upper chamber of a Transwell insert (PTFE membrane with 8 μm diameter pores, Corning). The lower chamber was filled with 1 mL of EBM-2 with 20% FBS with or without DEET. After 24 h, migrated cells were fixed with 4% paraformaldehyde for 15 min at room temperature. Cells were rinsed two times with washing buffer, stained with crystal violet (1 mg.mL^−1^ in 2% of ethanol, Sigma–Aldrich, C6158) for 10 min at room temperature and extensively washed with distilled water. Then, SDS 2% was added and incubated for 30 min at room temperature. Absorbance was then evaluated using a Mithras LB940 multimode microplate reader at 550 nm (Berthold Technologies).

### Western blot

After treatment, cells were homogenized and lysed. Proteins (30 μg) were separated on 10% SDS–polyacrylamide gel electrophoresis. Blots were probed with peNOS-Ser, peNOS-Thr (Cell Signaling, #9571, #9574), eNOS (BD Biosciences, 610297), p-FAK (Cell Signaling Technology, #3284) and VEGF antibodies (Santa Cruz Biotechnology, SC152). Monoclonal anti-β-actin antibody (Sigma–Aldrich, A5316) was used to visualize protein gel loading. The membranes were then incubated with the appropriate horseradish peroxidase-conjugated secondary antibody (Santa Cruz Biotechnology, SC2313 and SC2005). The protein–antibody complexes were detected by ECL plus (Thermo Scientific, #34096).

### Confocal microscopy

Once treated for 24 h with 10^−8 ^M or 10^−5 ^M DEET, HUVEC were fixed with 4% paraformaldehyde for 15 min at room temperature, permeabilized with 0.1% Triton X-100 in PBS and then blocked with 5% BSA in PBS for 1 h at room temperature. Cells were treated with a rabbit polyclonal p-FAK (Cell Signaling Technology, #3284) antibody in 5% BSA in PBS overnight at 4 °C. After washing with PBS, cells were treated with Alexa 488-conjugated goat anti-rabbit antibody (Molecular probe, 31210) in 5% BSA in PBS for 1 h at room temperature.

In another set of experiments, tetramethylrhodamine isothiocyanate-phalloidin (Phalloidin, Sigma–Aldrich, P1951) was used to label actin fibers. After treatments, cells were fixed with 4% paraformaldehyde and then stained with phalloidin (50 μg.mL^−1^) for 30 min at room temperature. After washing with PBS, cells were mounted and visualized with a confocal microscopy (CLMS 700, Zeiss, ZEN software).

### NO and superoxide anion (O_2_^−^) determinations by electron paramagnetic resonance (EPR)

Detection of NO production was performed using Fe^2+^ diethyldithiocarbamate (DETC, Sigma-Aldrich, D95503) as spin trap. Cells were treated with DEET for 24 h; medium was replaced with 250 μL of Krebs solution, then treated with 250 μL of colloid Fe(DETC)_2_ and incubated for 45 min at 37 °C. Cells were then scrapped and frozen in plastic tubes. NO detection was measured *in situ* by EPR. Values are expressed as amplitude of signal per protein concentration (unit.μg^−1^.μL of endothelial cell proteins).

For O_2_^−^ quantification, cells were allowed to equilibrate in deferoxamine-chelated Krebs-Hepes solution containing 1-hydroxy-3-methoxycarbonyl-2,2,5,5-tetramethylpyrrolidin (CMH; Noxygen, NOX-02) (500 μM), deferoxamine (25 μM), and DETC (5 μM) under constant temperature (37 °C) for 20 min. Cells were then scrapped and frozen in plastic tubes and analyzed by EPR spectroscopy. Values are expressed as percentage of control.

### Ca^2+^ response measurement

HUVEC were cultured on 8 wells μ-slides (Ibidi, Martinsried) and HEK293/M3 cells were cultured on a poly-L-lysine-coated 8 wells μ-slide (Ibidi, Martinsried) for 24 h before being washed with a Krebs solution (NaCl 119 mM, KCl 4.75 mM, MgSO_4_ 1.17, CaCl_2_ 2.5 mM, KH_2_PO_4_ 1.18, NaHCO_3_ 25 mM, Glucose 11.1 mM, Hepes 20 mM, pH = 7.40) and loaded with the molecular probe fluo-4 (3 μM, Molecular probe, F14204) for 30 min at 37 °C. Next, the cells were washed with Krebs solution and visualized with a confocal microscopy (CLMS 700 Zeiss, 488 nm/510 nm, ZEN software). After stabilization of the basal fluorescence, cells were stimulated with carbachol or ACh (10^−10 ^M to 10^−5 ^M) in presence or absence of DEET 1 min before. Images were acquired each second for 10 min using an X20 objective. Results are expressed in mean ± SEM of the ratio of maximal fluorescence divided by basal fluorescence.

### Docking

Primary sequence of M3 receptors are retrieved from Uniprot website (www.uniprot.com). Crystal structures of the muscarinic receptors are retrieved from the Protein Databank at www.rcsb.org.

Sequence alignment was performed with ClustalW^29^ as implemented at https://npsa-prabi.ibcp.fr/.

Surflex-Dock software^30^ as implemented in Sybyl-X 2.1 package (Tripos, MO, USA) was used for the docking simulation. The docking mode was GeomX with default options to have maximum accuracy. Prior to docking, the interaction cavity was defined with a “protomol” which represents the “negative of the cavity” in terms of acceptor, donor and hydrophobic groups. The protomol was generated according to the putative allosteric site defined by aligning 3D structure of M3 (PDB id: 4DAJ)^31^ with M2 (PDB id: 4MQT)^32^. To generate the protomol, these options were used: proto_thresh = 0.5 and proto_bloat = 0. Parameters used for the docking calculation were those by default: no flexibility on the protein, additional starting conformations per molecule multistart = 6, expand search grid grid_bloat = 6 Å, maximum conformations per fragment maxconfs = 20, maximum number of rotatable bonds per molecule maxrot = 100, allow ring flexibility in the molecule (+ring), soft grid treatment (+soft_box), pre- and post-docking minimization (+premin & +remin), fragment molecule (+frag), activate spin alignment method (+spinalign), density of search spindens = 9 for exhaustive accuracy, number of spins per alignment nspin = 12. Poses with a root mean square distance above div_rms = 0.5 Å are retained and a total of ndoc_final = 20 poses are output; for each pose, a calculated affinity expressed as −Log10(Kd) is given.

### Binding assay

Binding assay was performed by CEREP (Paris, France). Briefly, human recombinant M3 purified receptor (0.2 nM, CHO cell lines) is mixed with 0.2 nM [^3^H]-4-DAMP and the complex incubated for 60 min at room temperature in the absence or presence of DEET 10^−11^ M to 10^−4^ M. Nonspecific binding is determined in the presence of atropine (10 μM). The detection was realized by scintillation counting. The results are expressed as a percent inhibition of the control [^3^H]-4-DAMP specific binding. The standard reference compound is 4-DAMP, which is tested in each experiment at several concentrations to obtain a competition curve from which IC_50_ was calculated.

### Statistical analysis

Data are presented as mean ± SEM, *n* represents the number of experiments. For Ca^2+^ measurement, statistical analysis was performed by one-way ANOVA test. For other experiments, one-way non-parametric Kruskal-Wallis test followed by a Bonferroni correction were performed. *p* < 0.05 was considered to be statistically significant.

## Results

### DEET specifically stimulates endothelial cell to promote angiogenesis *in vitro* and *in vivo*

Tumor progression is a process that involves several cell types including cancer cells and endothelial cells. Cancer cells initiate tumor growth and endothelial cells are involved in angiogenesis, an essential process in the promotion of tumor growth and metastasis. The first step of the present study was to determine the cellular target that might be involved in the potential carcinogenic properties of DEET. We therefore analyzed the effect of DEET on proliferation of either U87MG cells or HUVECs. As shown in Table 1, DEET (10^−8 ^M or 10^−5 ^M) did not increase proliferation of cancer U87MG cells whereas it enhanced HUVEC proliferation when compared with non-treated cells. Since DEET is commonly applied onto the skin, the effects of DEET on B16F10 melanoma cell proliferation have been evaluated. No effect of DEET on the proliferation of B16F10 tumor cell line was observed (Supplemental Figure S1). To ensure the lack of cytotoxicity and apoptotic effects of DEET on HUVEC, viability and apoptosis measurements were performed for each concentration. Neither cytotoxic nor pro-apoptotic effects were observed in cells treated with DEET whereas actinomycin D (10^−6 ^M) increased HUVEC apoptosis (Supplementary Figure S2a,b). These results show that DEET promotes endothelial cell, but not cancer cell, proliferation.

**Figure 1:**
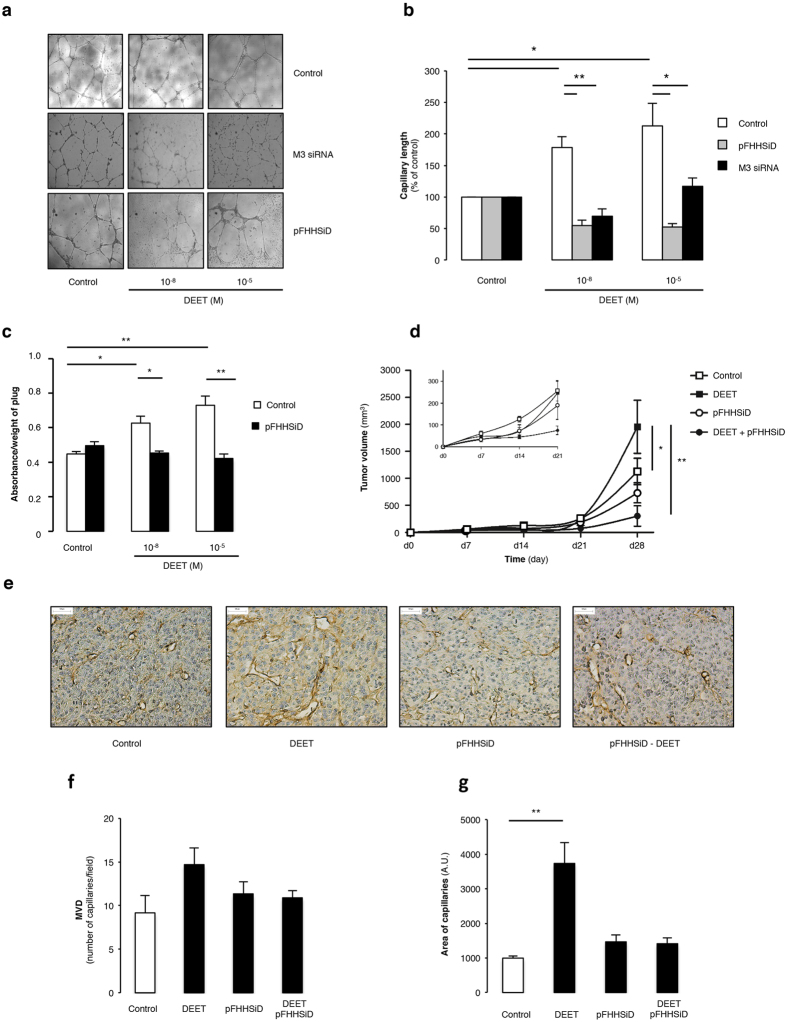
*In vitro* and *in vivo* pro-angiogenic properties of DEET. (**a**) Representative illustrations of HUVEC on ECMgel, treated during 24 h in absence or presence of DEET 10^−8 ^M or 10^−5 ^M with or without pFHHSiD 10^−7 ^M or silenced with specific M3 receptor siRNA. (**b**) DEET enhances *in vitro* capillary formation. Pharmacological blockade with pFHHSiD or silencing with specific siRNA against M3 muscarinic receptor were used. Reproducible data were obtained from four independent experiments. (**c**) DEET promotes *in vivo* neovascularization in ECMgel model. At day 14, quantitative measurement of hemoglobin was reported as absorbance (arbitray units)/weight of plugs. Hemoglobin content is increased in plugs containing mouse aortic-derived EC pretreated with DEET. Data were obtained from four independent experiments. Results are expressed at mean ± SEM. (**d**) DEET potentiates *in vivo* tumor growth. U87MG cells (10^6^) were injected subcutaneously into the flank of six-week old female nude Swiss mice. Mice were chronically treated with 10^−5 ^M DEET or its solvent (saline). The treatment was initiated the day following tumor cell injection and tumor dimensions were measured weekly. Pharmacological blockade with pFHHSiD prevents DEET-induced tumor growth. Data are expressed as tumor volume (mm^3^) and are presented as mean ± SEM (n = 6 *per* group). (**e**) DEET increases tumor-associated neovascularization. A representative paraffin section of tumor from each group (n = 6) is shown and reveals CD31 staining in brown. (**f**) Mean MVD (number of vessels/field) and mean area of capillaries (**g**) from tumors of mice treated with DEET and/or with pFHHSiD or saline are graphically represented. *p < 0.05; **p < 0.01 compared to control (Kruskal-Wallis with Bonferroni correction).

The effects of DEET were studied through *in vitro* angiogenesis analysis. DEET treatment with 10^−8 ^M or 10^−5 ^M increased capillary length formation in HUVEC (Fig. 1a,b, white columns). Compared to control, treatment with DEET concentrations ranging from 10^−14^ to 10^−5 ^M revealed that capillary length reaches a plateau at 10^−8 ^M DEET (Supplementary Figure S2). There was no significant difference in *in vitro* pro-angiogenic effects of HUVECs treated with DEET in the 10^−8 ^M and 10^−5 ^M concentration range.

To ascertain the effects of DEET on new vessel formation, we performed *in vivo* neovascularization studies. After 14 days, hemoglobin content was significantly increased in mice injected with endothelial cells that were previously cultured with 10^−8 ^M or 10^−5 ^M DEET (Fig. 1c, white columns).

In addition, DEET-induced neovascularization was studied in U87MG xenografted mice that were daily treated with DEET (intraperitoneal injection) at doses known to induce a plasma concentration of 10^−5 ^M, a concentration observed in exposed humans^23^. Detectable tumors (i.e. tumors >100 mm^3^) were observed 14 days after U87MG cell injection in mice. On day 28, tumor growth was significantly enhanced in mice treated with DEET compared to control mice (Fig. 1d, squares). Furthermore, CD31 staining of the tumors revealed that DEET significantly enhanced the area of capillaries but not microvascular density. (Fig. 1e–g).

### DEET induces pro-angiogenic effects through an M3 receptor-sensitive pathway and inhibits AChE activity

To determine the involvement of muscarinic receptors on DEET-induced proliferation, HUVEC were treated with 10^−8 ^M or 10^−5 ^M DEET in the presence of pFHHSiD (10^−7 ^M), a selective antagonist of M3 receptor. pFHHSiD completely prevented the proliferative properties of DEET for each concentration (Table 1).

Whether DEET increases capillary length was also investigated in HUVEC cells under these conditions. Similarly, pFHHSiD significantly prevented DEET-induced capillary length (Fig. 1a,b, grey columns). Furthermore, similar experiments were conducted with M3 siRNA. Although siRNA control was not effective in promoting the formation of capillaries, silencing of the M3 muscarinic receptor abolished the ability of DEET to increase capillary length (Fig. 1a,b, black columns).

In addition, *in vitro* vessel formation induced by 10^−8 ^M or 10^−5 ^M DEET and *in vivo* neovascularization were completely prevented by pFHHSiD (Fig. 1c,d, circles). These results revealed that DEET promotes angiogenesis through a pathway sensitive to a selective antagonist of M3 receptor or a M3 siRNA.

**Figure 2:**
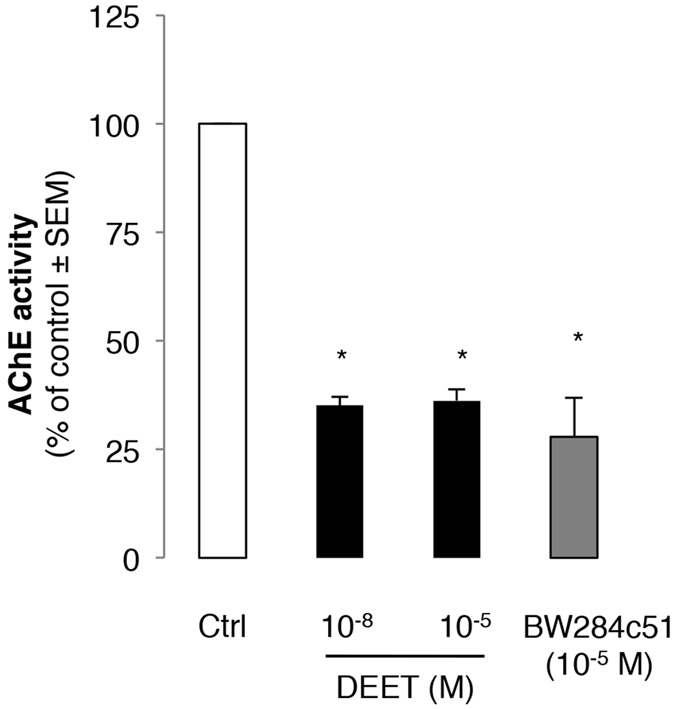
DEET decreases acetylcholinesterase activity on HUVEC. Both DEET concentrations (10^−8 ^M and 10^−5 ^M) decrease acetylcholinesterase activity on HUVEC. BW284c51 (10^−5 ^M) is used as control as acetylcholinesterase inhibitor. Results are expressed as percentage of AChE activity on HUVEC treated with both concentrations of DEET compared to cells treated with solvent. Values are expressed at means ± SEMs from six independent experiments. *p < 0.05 compared to control (Kruskal-Wallis with Dunn’s multiple comparison test).

AChE is a molecular target of DEET. Drugs, such as donepezil, act as an AChE inhibitor and are able to promote angiogenesis both in *in vitro* EC and in a murine ischemic hind limb model^14^. We tested the effect of both concentrations of DEET on AChE enzyme activity in HUVEC. Low (10^−8 ^M) and high (10^−5 ^M) concentrations of DEET decreased endothelial AChE activity compared to the control condition (Fig. 2).

### DEET stimulates endothelial cell migration, adhesion as well as expression of known endothelial markers of angiogenesis

**Figure 3:**
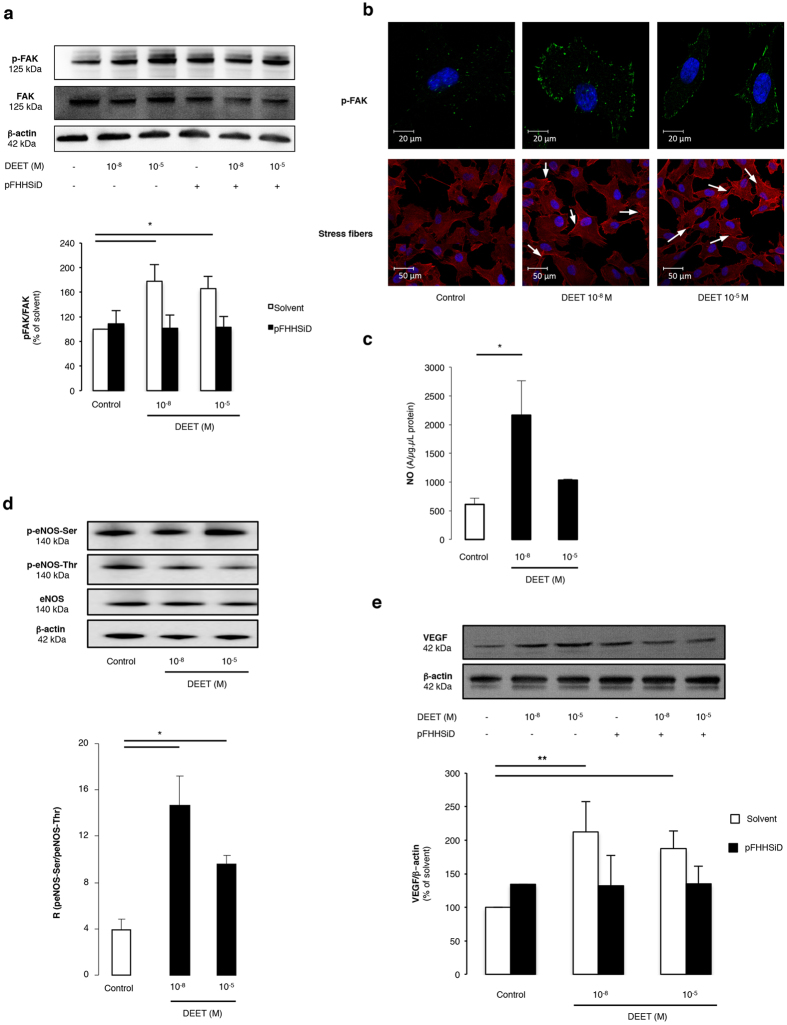
Cellular processes and molecular signaling pathways involved in DEET-induced pro-angiogenic effect. (**a,b**) Western blotting and immunofluorescence staining of HUVEC for p-FAK show an activation of FAK pathway after treatment with DEET for 24 h. Data are obtained from four independent blots. Horizontal bar = 20 μm. To highlight FAK phosphorylation and actin stress fibers, HUVEC were stained with a p-FAK antibody or rhodamine-labelled phalloidin and visualized by confocal microscopy. DEET induces the formation of stress fibers (white arrow). Horizontal bar = 50 μm. (**c**) Quantification of the amplitude of the NO-Fe(DETC)_2_ complex signal by electron paramagnetic (EPR) in HUVEC reveals a significant increase of NO production in cells treated with 10^−8^ or 10^−5 ^M DEET compared with control cells. Values are expressed as units per microgram per microliter of protein in the samples. Results are from four independent experiments. (**d**) Western blot revealed eNOS expression and phosphorylation of Ser-1177 (activator site) and of Thr-495 (inhibitory site). β-actin control is included. DEET (10^−8 ^M and 10^−5 ^M) increases the ratio between p-eNOS-Ser and p-eNOS-Thr. Results are means ± SEMs from four independent experiments. (**e**) Western blot shows VEGF protein expression after treatment with DEET (10^−8 ^M and 10^−5 ^M). Ratio between VEGF expression and β-actin expression shows that 10^−8 ^M and 10^−5 ^M DEET increase VEGF expression. Data are representative of four separate blots. Results are means ± SEM. *p < 0.05; **p < 0.01 compared to control (Kruskal-Wallis with Dunn’s multiple comparison test).

10^−8 ^M and 10^−5 ^M concentration of DEET in HUVECs significantly enhanced endothelial cell migration and adhesion compared to non-treated cells (Table 2). These effects were completely prevented by pFHHSiD (Table 2). We then investigated the expression and the activation of proteins involved in cell migration and/or adhesion. The two concentrations of DEET increased the phosphorylation of focal adhesion kinase (FAK) without modifying its expression (Fig. 3a). Furthermore, pFHHSiD prevented DEET-induced FAK phosphorylation (Fig. 3a). Likewise, phalloidin staining confirmed that DEET is able to induce formation of stress fibers in HUVEC (Fig. 3b).

Nitric oxide (NO) is involved in the angiogenic process^33^, therefore, NO production in HUVEC stimulated with DEET was investigated. Endothelial NO production increased with DEET treatment compared to control cells, but this only reached significance in the 10^−8 ^M dose (Fig. 3c). To determine the molecular changes governing DEET-induced NO production, the expression and activation of eNOS were analyzed by Western blot. Although treatment with DEET did not modify eNOS expression, it significantly increased eNOS phosphorylation on its activator site (Ser-1177) while decreasing it at the inhibitor site (Thr-495) compared to non-treated cells (Fig. 3d). In addition, after normalization of the amount of phosphorylated eNOS to total amount of the enzyme, the ratio of phosphorylated eNOS at the activator and inhibitor sites was calculated. This ratio was significantly increased in HUVEC treated with each concentration of DEET compared with non-treated cells (Fig. 3d).

NO bioavailability depends on superoxide anion (O_2_^−^) production in endothelial cells. To ensure that O_2_^−^ did not reduce NO bioavailability, the quantification of O_2_^−^ production was assessed by EPR and revealed that neither 10^−8 ^M (99.8 ± 8.9%) nor 10^−5 ^M (80.4 ± 11.4%) DEET concentrations modified O_2_^−^ production compared with control cells (100%).

Finally, the expression of one of the central pro-angiogenic factors, VEGF, was analyzed by Western blot. DEET significantly enhanced VEGF expression which was completely prevented with pFHHSiD (Fig. 3e).

### DEET acts as an allosteric modulator of the M3 receptor leading to subtle modulation of calcium signaling

Activation of the M3 receptor leads to Ca^2+^ release from inositol 1,4,5-trisphosphate (IP3)-sensitive stores and endothelial calcium signals, which promotes angiogenesis^34^,^35^.

**Figure 4:**
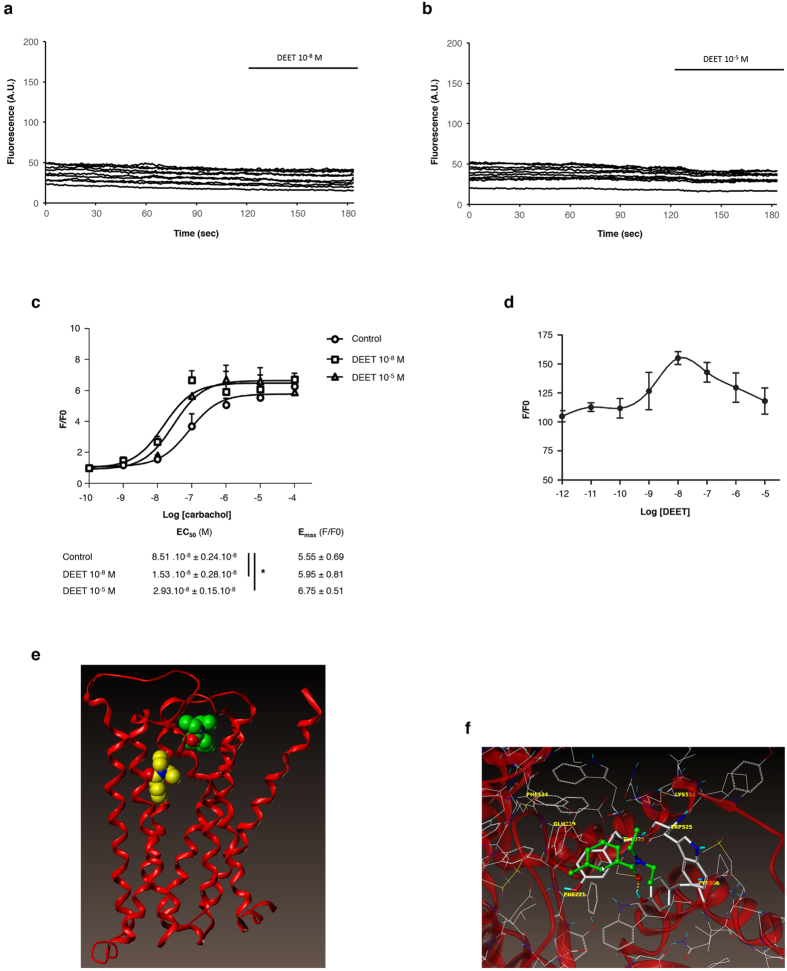
DEET is a modulator of M3 receptor. (**a,b**) Time course of the fluorescence of 10 HEK/M3 cells loaded with fluo-4. DEET does not increase the fluorescence at the two used concentrations. (**c**) Concentration-response curves of different carbachol independent concentrations on [Ca^2+^]*i* increase on HEK/M3 in presence or in absence of DEET (10^−8 ^M or 10^−5 ^M). The peak response of [Ca^2+^]*i* induced by each agonist concentration is normalized in fold (F/F0). Each dot in the curve represents the mean ± SEM of three to six independent experiments. *p < 0.05 compared to control (ANOVA). (**d**) Modulation of the Ca^2+^ response in HEK/M3 induced by EC_50_ of carbachol (10^−8 ^M) *versus* the concentration of DEET. This response is increased for 10^−8 ^M of DEET. (**e**) M3 backbone is represented in red ribbon. Red balls correspond to oxygen atoms and blue ones to nitrogen. Carbon atoms are in yellow for DEET docked into the orthosteric site and in green for DEET docked into the allosteric site. (**f**) DEET in the putative allosteric site. Carbon atoms are in white for the protein and green for DEET; nitrogen are in blue, oxygen in red, sulfur in yellow and hydrogen in cyan. DEET is displayed in ball and stick fashion and residues of M3 interacting directly with DEET are highlighted in stick fashion. Dash yellow lines correspond hydrogen bonds.

Since the effect of DEET was sensitive to both pharmacological blockade and silencing of M3 receptor, we tested the hypothesis that it may increase calcium signaling. Surprisingly, DEET was not able to increase cytosolic calcium both in HUVEC (data not shown) and HEK/M3 (Fig. 4a,b) for the two concentrations used.

To better characterize the mechanism by which DEET acts on the M3 receptor and due to heterogeneity of carbachol-induced Ca^2+^ response in endothelial cells^36^, the effects of DEET on carbachol-induced calcium signaling were evaluated in the same manner as in HEK/M3. As shown in Fig. 4c, carbachol induced a concentration-dependent increase in cytosolic calcium, the maximum being achieved at 1 μM (EC_50_: 8.51 × 10^−8^ ± 0.24 × 10^−8 ^M). Whatever the concentration of DEET used, it was able to induce a leftward shift of the calcium response induced by carbachol with an EC_50_ equal to 1.53 × 10^−8^ ± 0.28 × 10^−8^ and 2.93 × 10^−8^ ± 0.15 × 10^−8 ^M, p < 0.05, for 10^−8 ^M and 10^−5 ^M DEET, respectively (Fig. 4c). Moreover, DEET modulated the Ca^2+^ response induced by 10^−8 ^M of carbachol, which corresponds to EC_50_ of carbachol in these conditions, in a window of concentrations beginning at 10^−8 ^M and extended to 10^−7 ^M (Fig. 4d). These results suggest that DEET, *via* allosteric modulation of the M3 receptor, increases the carbachol-induced calcium rise.

To confirm this hypothesis, docking assay was performed through a docking model of the M3 receptor derived from the structure of PDB (PDB id: 4DAJ^31^) (Fig. 4e,f). As the homology of rat and human M3 receptors is very high (98% of homology, cf. sequence alignment in Supplementary Figure S3) and because the non-conserved residues are located outward of the receptor, the 4DAJ structure was used as our docking model.

The rat M3 receptor (PDB id: 4DAJ^31^) showed a well-defined orthosteric site and revealed the presence of a potential allosteric site (Fig. 4e,f) as compared to the crystal structure of M2 receptor^32^.

Thus a docking study was performed to explore the possibility that DEET binds at this putative allosteric site instead of the orthosteric site. The «best pose» ranked by the affinity score had a calculated affinity −Log10(Kd) of 5.04 and 4.35, respectively, for the orthosteric and allosteric site (Fig. 4e,f). In the same manner, a binding assay was performed to confirm that DEET does not act on the orthosteric site of M3 receptor. As shown in Table 3, for concentrations ranging from 10^−11 ^M to 10^−4 ^M, DEET was not able to displace ^3^[H] 4-DAMP on M3 receptor.

## Discussion

Epidemiological studies report different side effects of the use of DEET including neurologic side effects^37^, seizures^38^ and cancer^6^,^39^, the last of which is a pathophysiological situation in which angiogenesis plays an important role^40^. Angiogenesis is critical for tumor development, and it is considered as a pre-requisite for the rapid expansion of tumor cells associated with formation of macroscopic tumors^16^. In the present study, we demonstrate that DEET acts directly on the endothelial cells to promote cellular processes leading to *in vitro* angiogenesis. Furthermore, we reveal that DEET is able to favor *in vivo* tumor growth through an enhancement of neovascularization without inducing U87MG glioblastoma-astrocytoma cell proliferation. In endothelial cells, DEET activates different steps leading to angiogenesis including migration and adhesion associated with phosphorylation of FAK, enhanced expression of VEGF and activation of NO pathway by a mechanism sensitive to pharmacological blocker or silencing of M3 receptor. Importantly, by using calcium signaling, binding and docking assays, this study highlights a mechanism underlying effects of DEET *via* an allosteric modulation of M3 receptor.

A correlation has been established between plasma levels of DEET (10^−5 ^M)^23^ and the risk of developing cancer^39^. Furthermore, regular use of DEET as a mosquito repellent has resulted in this molecule being present in the air, rain and rivers^41^,^42^. As a consequence, concentrations of DEET as high as 10^−8 ^M^24^ have been observed in drinking water. Once introduced in the blood circulation, DEET can remain for an extended period of time which implicates EC to be one of the primary targets of the toxicity of these compounds. Thus, to assess the impact of these concentrations on angiogenic processes, the present study has been performed using the maximum concentration found in surface water and wastewater and the maximum plasma concentrations^42^.

Because U87MG tumor epithelial cells are classically used as an experimental model of glioblastoma to investigate tumor angiogenesis *in vivo*^43^,^44^,^45^, the tumor properties of DEET have been assessed on this model. We found that DEET does not induce U87MG tumor cell proliferation whereas it favors proliferation, migration and adhesion of endothelial cells. Furthermore, according to immunostaining data, the present study confirms that DEET increases tumor promotion by enhancing neovascularization. Indeed, we report pro-angiogenic properties through *in vitro* studies in which DEET enhances FAK phosphorylation and induces the formation of stress fibers. These findings suggest that DEET plays a significant role in migration and adhesion of endothelial cells. These effects are associated with an increase of NO production and VEGF expression, two cellular mediators playing an essential role in the regulation of angiogenesis^16^,^46^. VEGF is a key growth factor that is highly upregulated in tumors and controls anarchic neovascularization^47^. In the present study, we reported that DEET-induced VEGF overexpression is dependent on activation of M3 muscarinic receptors. Because VEGFR-2 is the main VEGF receptor involved in cellular processes leading to *in vitro* angiogenesis^48^, we suggest that cellular processes involved in DEET-induced angiogenesis are consequences of VEGF induction rather than additional effects.

The molecular targets of DEET in insects that explain its repellent activity are still debated. Potential mechanisms are inhibition of acetylcholinesterase (AChE)^8^, modulation of muscarinic receptors^49^ or the well-conserved ionotropic receptor Ir40a, odorant receptors (OR) and gustatory receptors (GR)^7^,^50^,^51^,^52^,^53^,^54^. Among these targets, it has been reported that some inhibitors of human AChE, like donepezil, rivastigmine or galantamine have pro-angiogenic properties in *in vitro* HUVEC cultured on ECMgel like in chick chorioallantoic membrane and/or in an ischemic hind limb model^14^,^55^.

In the present study, we reveal that both concentrations of DEET (10^−8 ^M and 10^−5 ^M) significantly inhibited endothelial AChE activity, as has been demonstrated by Corbel *et al*.^8^. Inhibitors of AChE and acetylcholine itself can promote *in vitro* angiogenesis through an atropine-sensitive mechanism^14^. Moreover, it has recently been reported that HUVEC possess an autocrine non-neuronal cholinergic system involved in the regulation of angiogenesis^15^. Thus, in the present study, we show that the ability of DEET to increase angiogenic processes *in vitro* and *in vivo* was abrogated after pharmacological blockade or silencing of the M3 receptor. These results could explain the pro-angiogenic effect of DEET at both concentrations.

Indeed, although the M3 muscarinic receptor is expressed in the nervous system^56^, it is now known that this cholinergic receptor is also expressed in several non-innervated tissues such as endothelial^10^ or tumor cells, as well as human U87MG glioblastoma cells^57^. Interestingly, up-regulation of tumor neovascularization by DEET is significantly prevented by pFHHSiD. These results are partly explained by the analysis of both tumor MVD and area of capillaries, which show a decrement in the number of vessels in tumors treated simultaneously by DEET and pFHHSiD. These findings confirm previous studies performed on lung and colon cancer cells that expressed a cholinergic autocrine loop. In these cellular models, acetylcholine secreted by cancer cells or neighboring cells interacts with M3 muscarinic receptors to stimulate tumor growth^58^ by increasing angiogenesis.

MVD may reflect the degree of angiogenesis in solid tumors. The present study reports that DEET increased MVD through a pFHHSiD sensitive pathway but in a non-significant manner. Microvessel growth is anarchic in solid tumors, producing disorganized and tortuous vessels that can vary in size^59^. Moreover, depending on the maturity of blood vessels, their spatial heterogeneity and their inner diameter, MVD does not systematically correlate with functional vascularity. Thus, the quality of MV must be also appreciated by the area of vessels that reflect their diameter, as described in the present work.

Additionally, angiogenesis processes involve endothelial cell activation as depicted by increases in intracellular calcium^34^,^35^. Due to a heterogeneity of carbachol-induced Ca^2+^ response in endothelial cell^36^, the effects of DEET on carbachol-induced calcium signaling were evaluated in HEK/M3. In the present study, we report that DEET is not able to increase cytosolic calcium both in HUVEC and HEK/M3 cell lines at the two used concentrations, but it induces a leftward shift of the calcium response induced by carbachol on HEK/M3. These data lead us to hypothesize that DEET behaves as an allosteric modulator of M3 receptor.

The docking model of the M3 receptor used in the present study defined both orthosteric and allosteric sites. The values of the calculated affinity of DEET at both sites were not discriminative. Therefore we cannot rule out that DEET may bind to M3 at both sites or preferably on one of the two sites. DEET may thus have more than one interaction mode with M3, certainly depending on its concentration. Other works by Kruse *et al*.^32^ on the M2 receptor and the simulation performed by Abd-Ella *et al*.^49^ on M1/M3 receptors did support the existence of an allosteric site in human M3 receptor. At low concentration (10^−8 ^M), DEET interacts with high affinity with M1/M3 mAChR allosteric sites, whereas at high concentration (>10^−6 ^M), DEET interacts with a very low affinity^49^.

To discard the hypothesis that DEET acts directly on the orthosteric site of M3 receptor to mediate its effect, a binding assay between DEET and M3 receptor was performed. DEET is not able to displace the binding of the labeled specific M3 receptor antagonist ^3^[H] 4-DAMP. Altogether, the most likely hypothesis is that DEET acts as an allosteric modulator of the M3 receptor allowing the potentiating effect on calcium signaling on endothelial cells and consequently angiogenesis (*in vitro* and *in vivo*) and tumor growth *in vivo*.

**Figure 5:**
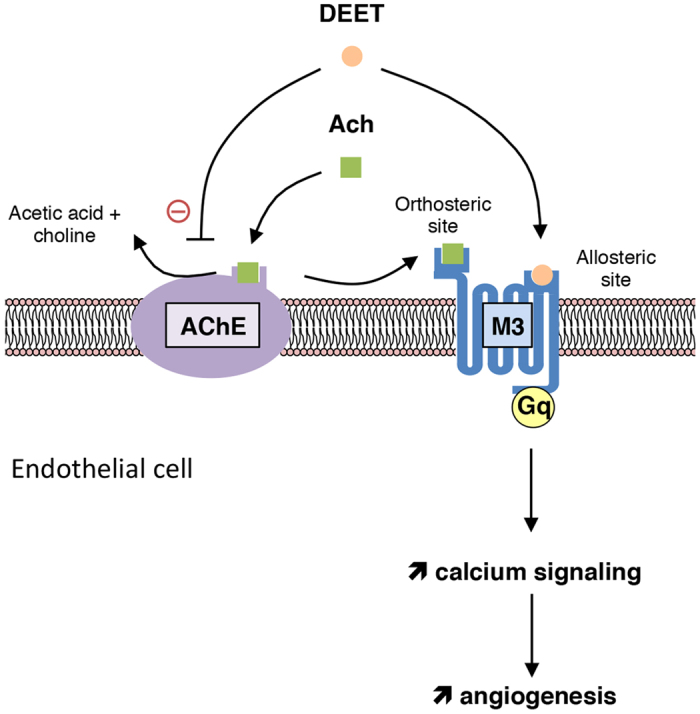
Summary of the DEET-induced angiogenesis mechanism. DEET inhibits AChE, the enzyme responsible for the degradation of acetylcholine in choline and acetic acid, and binds an allosteric site of M3 leading to activation of angiogenic process.

DEET inhibits AChE, thereby increasing acetylcholine bioavailability and binding to its M3 receptor, while strengthening proangiogenic effects by an allosteric modulation (Fig. 5).

Altogether, these data suggest pro-angiogenic properties of DEET in human health. More importantly and for the first time, these data show that an allosteric modulator of M3 muscarinic receptor subtype can promote both *in vitro* and *in vivo* angiogenesis. Considering the environmental pollution induced by DEET worldwide and its presence in drinking water sources, new water treatment technologies are needed for its elimination. Finally, risk assessment of DEET should now be implemented in humans in order to provide safe conditions of use of this insect repellent.

## Additional Information

**How to cite this article**: Legeay, S. *et al*. The insect repellent *N*,*N*-diethyl-*m*-toluamide (DEET) induces angiogenesis *via* allosteric modulation of M3 muscarinic receptor in endothelial cells. *Sci. Rep.* **6**, 28546; doi: 10.1038/srep28546 (2016).

## Supplementary Material

Supplementary Information

## Figures and Tables

**Table 1:** Endothelial cells are the main targets of DEET.

Proliferation (% of control ± SEM)	DEET 10^−8 ^M	DEET 10^−5 ^M	pFHHSiD 10^−7 ^M DEET 10^−8 ^M	pFHHSiD 10^−7 ^M DEET 10^−5 ^M
U87MG	98.21 ± 1.59	97.18 ± 1.28	/	/
HUVEC	113.55 ± 3.16*	112.03 ± 2.90*	102.79 ± 5.41	100.82 ± 5.23

CyQUANT assay reveals that both concentrations of DEET (10^−8 ^M and 10^−5 ^M) do not increase proliferation of U87MG cell line while increasing HUVEC one after 24 h treatment. The selective M3 muscarinic receptor antagonist pFHHSiD prevents the DEET-induced endothelial proliferation. Results are expressed at mean ± SEM; *p < 0.05 compared to control (Kruskal-Wallis with Dunn’s multiple comparison test).

**Table 2:** DEET increases HUVEC adhesion and migration through a pFHHSiD sensitive pathway.

	DEET 10^−8 ^M	DEET 10^−5 ^M	pFHHSiD 10^−7 ^M DEET 10^−8 ^M	pFHHSiD 10^−7 ^M DEET 10^−5 ^M
Adhesion (% of control ± SEM)	139.85 ± 11.94*	142.45 ± 13.89*	94.81 ± 6.07	84.88 ± 7.58
Migration (% of control ± SEM)	122.08 ± 3.92*	133.75 ± 8.21*	47.70 ± 2.49*	79.74 ± 3.71*

Values are expressed as percentage of solvent. Results are expressed at means ± SEMs from four independent experiments. *p < 0.05 compared to control (Kruskal-Wallis with Dunn’s multiple comparison test).

**Table 3:** Binding assay of DEET on human muscarinic M3 receptor.

Concentration of DEET (M)	10^−11^	10^−10^	10^−9^	10^−8^	10^−7^	10^−6^	10^−5^	10^−4^
% of control specific binding	104.3	106.4	104.7	102.1	106.6	113.5	112.2	114.4

Results are from CEREP manufactory and are expressed at mean of the % of control specific binding of one duplicate.
