# Supplementary material for: The insect repellent N,N-diethyl-m-toluamide (DEET) induces angiogenesis via allosteric modulation of the M3 muscarinic receptor in endothelial cells
Source: Sci Rep. 2016 Jun 27;6:28546. doi: 10.1038/srep28546 (PMC4921870; doi:10.1038/srep28546)
Supplement: Supplementary Information [file srep28546-s1.pdf]

The insect repellent *N,N*-diethyl-*m*-toluamide (DEET) induces angiogenesis *via* allosteric modulation of M3 muscarinic receptor in endothelial cells

Samuel Legeay[1], Nicolas Clere[1], Grégory Hilairet[1], Quoc-Tuan Do[2], Philippe Bernard[2], Jean-François Quignard[3], Véronique Apaire-Marchais[4], Bruno Lapied[4], Sébastien Faure[1].

[1]Inserm U1063 - Stress oxydant et pathologies métaboliques, LUNAM Université, Angers, France

[2]GreenPharma S.A.S. 3 allée du titane, 45100 Orléans, France.

[3]Inserm U1045 - Centre de Recherche Cardio-Thoracique de Bordeaux, Université de Bordeaux, France.

[4]Laboratoire des Récepteurs et Canaux Ioniques Membranaires (RCIM), UPRES-EA 2647, USC INRA 1330, Angers, France.

Address for correspondence:
Sébastien Faure, PharmD, PhD
UMR INSERM 1063 – Université d'Angers
Institut de biologie en santé
Rue des Capucins
49045 Angers Cedex
France
sebastien.faure@univ-angers.fr
Tel: +33 2 41 22 67 40

Supplementary data

Figure S1. DEET does not modify proliferation of B16F10 melanoma cell line

CyQUANT® assay reveals that both concentrations of DEET (10^{-8} M and 10^{-5} M) do not increase proliferation of B16F10 cell line. Results are expressed at mean ± SEM ; *$p < 0.05$ compared to control (Kruskal-Wallis with Dunn's multiple comparison test).

Figure S2. DEET does not induce neither cytotoxicity nor apoptosis in HUVEC

(a) *In vitro* cell viability was assessed by colorimetric analysis of MTT reduction after a treatment with 10^{-8} M or 10^{-5} M DEET for 24 h. (b) Flow cytometric DNA content histograms (fluorescence in arbitrary units, AU) of HUVEC exposed to 10^{-8} or 10^{-5} M DEET for 24 h, show that DEET does not induce apoptosis. Actinomycin D (10^{-6} M) was used as positive control. *$p<0.05$; **$p<0.01$ compared to control (Kruskal-Wallis with Dunn's multiple comparison test).

Figure S3. *In vitro* capillary formation at increasing concentrations of DEET

HUVEC were cultured on ECMgel® and treated for 24h with concentrations ranging from 10^{-14} M to 10^{-5} M DEET. 10^{-8}M DEET allows reaching the plateau of capillaries formation. Reproducible data are obtained from five to six independent experiments. *$p<0.05$; ***$p<0.001$ compared to control (Kruskal-Wallis with Dunn's multiple comparison test).

Figure S4. Sequence alignment of rat and human M3 receptor

Supplemental data
Figure S1

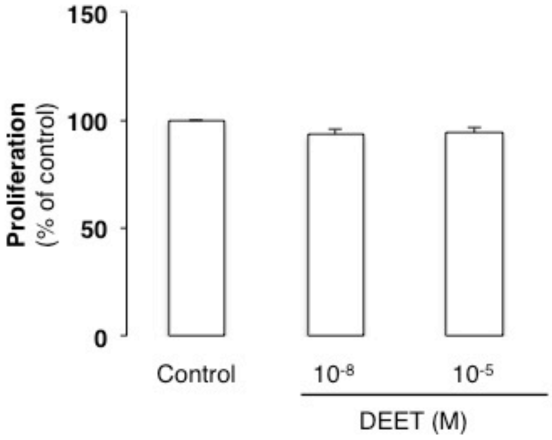

Supplemental data
Figure S2

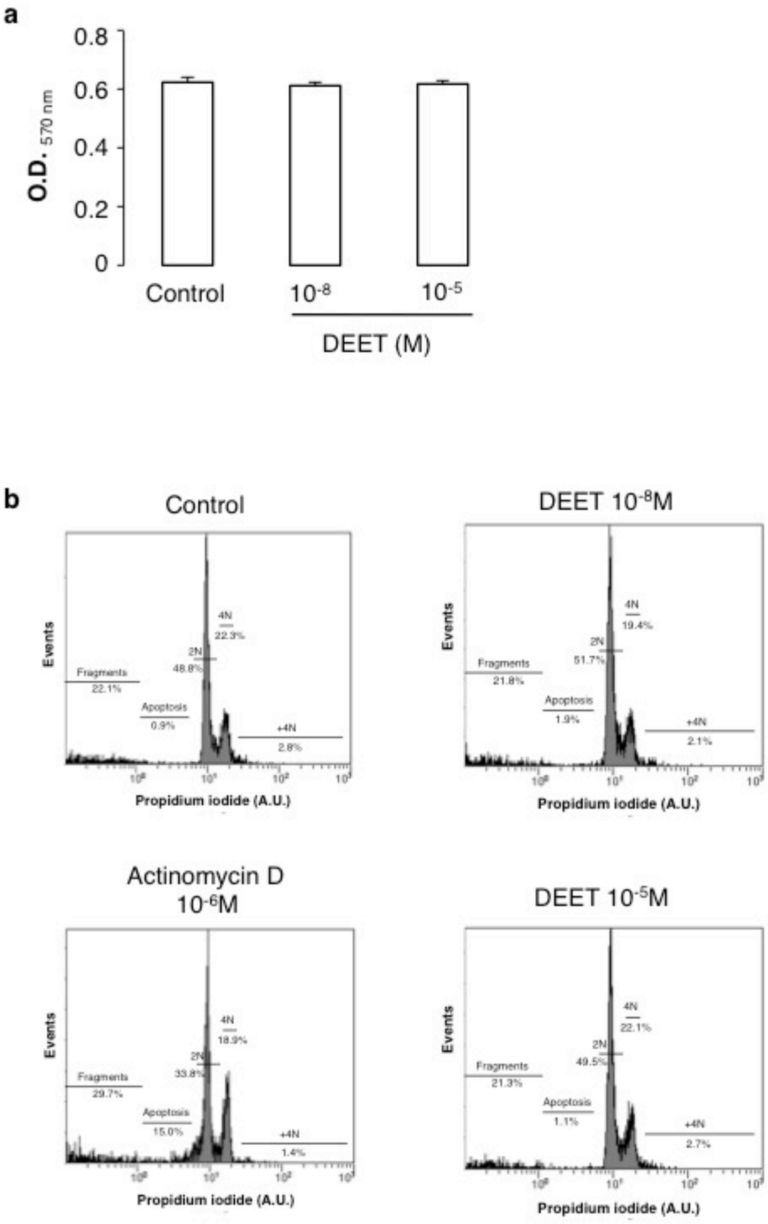

Supplemental data
Figure S3

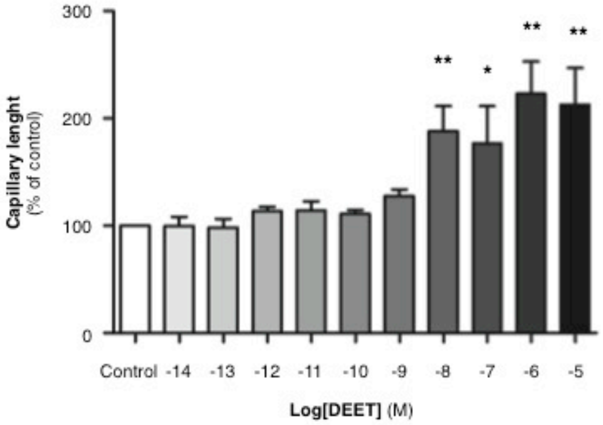

Supplemental data
Figure S4

```
ACM3_HUMAN   MTLHNNSTTSPLFPNISSSWIHSPSDAGLPPGTVTHFGSYNVSRAAGNFSSPDGTTDDPL
ACM3_RAT     MTLHSNSTTSPLFPNISSSWVHSPSEAGLPLGTVTQLGSYNISQETGNFSSND-TSSDPL
             ****.***************:****:**** ****::****:*: :***** * *:.***
Prim.cons.   MTLH2NSTTSPLFPNISSSW2HSPS2AGLP2GTVT22GSYN2S222GNFSS2DGT22DPL

                     70        80        90       100       110       120
                      |         |         |         |         |         |
ACM3_HUMAN   GGHTVWQVVFIAFLTGILALVTIIGNILVIVSFKVNKQLKTVNNYFLLSLACADLIIGVI
ACM3_RAT     GGHTIWQVVFIAFLTGFLALVTIIGNILVIVAFKVNKQLKTVNNYFLLSLACADLIIGVI
             ****:***********:**************:****************************
Prim.cons.   GGHT2WQVVFIAFLTG2LALVTIIGNILVIV2FKVNKQLKTVNNYFLLSLACADLIIGVI

                    130       140       150       160       170       180
                      |         |         |         |         |         |
ACM3_HUMAN   SMNLFTTYIIMNRWALGNLACDLWLAIDYVASNASVMNLLVISFDRYFSITRPLTYRAKR
ACM3_RAT     SMNLFTTYIIMNRWALGNLACDLWLSIDYVASNASVMNLLVISFDRYFSITRPLTYRAKR
             *************************:**********************************
Prim.cons.   SMNLFTTYIIMNRWALGNLACDLWL2IDYVASNASVMNLLVISFDRYFSITRPLTYRAKR

                    190       200       210       220       230       240
                      |         |         |         |         |         |
ACM3_HUMAN   TTKRAGVMIGLAWVISFVLWAPAILFWQYFVGKRTVPPGECFIQFLSEPTITFGTAIAAF
ACM3_RAT     TTKRAGVMIGLAWVISFVLWAPAILFWQYFVGKRTVPPGECFIQFLSEPTITFGTAIAAF
             ************************************************************
Prim.cons.   TTKRAGVMIGLAWVISFVLWAPAILFWQYFVGKRTVPPGECFIQFLSEPTITFGTAIAAF

                    250       260       270       280       290       300
                      |         |         |         |         |         |
ACM3_HUMAN   YMPVTIMTILYWRIYKETEKRTKELAGLQASGTEAETENFVHPTGSSRSCSSYELQQQSM
ACM3_RAT     YMPVTIMTILYWRIYKETEKRTKELAGLQASGTEAEAENFVHPTGSSRSCSSYELQQQGV
             ************************************:*********************.:
Prim.cons.   YMPVTIMTILYWRIYKETEKRTKELAGLQASGTEAE2ENFVHPTGSSRSCSSYELQQQ22

                    310       320       330       340       350       360
                      |         |         |         |         |         |
ACM3_HUMAN   KRSNRRKYGRCHFWFTTKSWKPSSEQMDQDHSSSDSWNNNDAAASLENSASSDEEDIGSE
ACM3_RAT     KRSSRRKYGRCHFWFTTKSWKPSAEQMDQDHSSSDSWNNNDAAASLENSASSDEEDIGSE
             ***.*******************:************************************
Prim.cons.   KRS2RRKYGRCHFWFTTKSWKPS2EQMDQDHSSSDSWNNNDAAASLENSASSDEEDIGSE

                    370       380       390       400       410       420
                      |         |         |         |         |         |
ACM3_HUMAN   TRAIYSIVLKLPGHSTILNSTKLPSSDNLQVPEEELGMVDLERKADKLQAQKSVDDGGSF
ACM3_RAT     TRAIYSIVLKLPGHSSILNSTKLPSSDNLQVSNEDLGTVDVERNAHKLQAQKSMGDGDNC
             ***************:***************.:*:** **:**:*.*******:.**.. 
Prim.cons.   TRAIYSIVLKLPGHS2ILNSTKLPSSDNLQV22E2LG2VD2ER2A2KLQAQKS22DG222

                    430       440       450       460       470       480
                      |         |         |         |         |         |
ACM3_HUMAN   PKSFSKLPIQLESAVDTAKTSDVNSSVGKSTATLPLSFKEATLAKRFALKTRSQITKRKR
ACM3_RAT     QKDFTKLPIQLESAVDTGKTSDTNSSADKTTATLPLSFKEATLAKRFALKTRSQITKRKR
              *.*:************.****.***..*:******************************
Prim.cons.   2K2F2KLPIQLESAVDT2KTSD2NSS22K2TATLPLSFKEATLAKRFALKTRSQITKRKR

                    490       500       510       520       530       540
                      |         |         |         |         |         |
ACM3_HUMAN   MSLVKEKKAAQTLSAILLAFIITWTPYNIMVLVNTFCDSCIPKTFWNLGYWLCYINSTVN
ACM3_RAT     MSLIKEKKAAQTLSAILLAFIITWTPYNIMVLVNTFCDSCIPKTYWNLGYWLCYINSTVN
             ***:****************************************:***************
Prim.cons.   MSL2KEKKAAQTLSAILLAFIITWTPYNIMVLVNTFCDSCIPKT2WNLGYWLCYINSTVN

                    550       560       570       580       590
                      |         |         |         |         |
ACM3_HUMAN   PVCYALCNKTFRTTFKMLLLCQCDKKKRRKQQYQQRQSVIFHKRAPEQAL
ACM3_RAT     PVCYALCNKTFRTTFKTLLLCQCDKRKRRKQQYQQRQSVIFHKRVPEQAL
             **************** ********:******************.*****
Prim.cons.   PVCYALCNKTFRTTFK2LLLCQCDK2KRRKQQYQQRQSVIFHKR2PEQAL
```

Alignment data :

Alignment length : 590
Identity (*) : 541 is 91.69 %
Strongly similar (:) : 26 is 4.41 %
Weakly similar (.) : 15 is 2.54 %
Different : 8 is 1.36 %
Sequence 0001 : ACM3_HUMAN (590 residues).
Sequence 0002 : ACM3_RAT (589 residues).
